# The Efficacy of Transcarotid Artery Revascularization With Flow Reversal System Compared to Carotid Endarterectomy: A Systematic Review and Meta-Analysis

**DOI:** 10.3389/fcvm.2021.695295

**Published:** 2021-11-19

**Authors:** Jianfeng Gao, Zhong Chen, Lei Kou, Hanfang Zhang, Yaoguo Yang

**Affiliations:** Department of Vascular, Beijing Anzhen Hospital, Capital Medical University, Beijing, China

**Keywords:** carotid endarterectomy (CEA), carotid stenosis, stroke, meta-analysis, transcarotid artery revascularization (TCAR)

## Abstract

**Background:** Carotid artery stenosis has long been a critical cause of stroke and death, and it can seriously affect the life quality. Transcarotid artery revascularization (TCAR) and carotid endarterectomy (CEA) are both feasible therapies for this disease. This systematic review and meta-analysis aim to evaluate if the efficacy of the two approaches is comparable.

**Methods:** Clinical studies up to March 2021 were searched through PubMed, Embase, and Scopus from a computer. The screening process was designed according to the Preferred Reporting Items for Systematic Reviews and Meta-Analysis (PRISMA) statement. Newcastle-Ottawa Scale (NOS) was used for methodological quality assessment of works of literature meeting the inclusion criteria, and Review Manager 5.4 was used for data synthesis. The I^2^ statistic was performed to measure the heterogeneity, and M-H/I-V fixed or random model was utilized depending on the I^2^ value. The evidence evaluation was accomplished based on grades of recommendation, assessment, development, and evaluation (GRADE) online tool.

**Results:** A total of 14,200 subjects (six comparative studies) were finally included in this pooled study. There is no statistical discrepancy between the two treatments on reducing stroke/death/myocardial infarction (odds ratio [OR] 0.85, 95% CI 0.67–1.07), stroke (OR 1.03, 95% CI 0.77–1.37), or death (OR 1.14, 95% CI 0.67–1.94). Besides, TCAR is associated with a lower incidence of myocardial infarction (*P* = 0.004), cranial nerve injury (*P* < 0.00001), and shorter procedure time (*P* < 0.00001) than CEA among the overall cohort.

**Conclusions:** TCAR is a rapidly developing treatment that reaches a comparable prognosis to CEA and significantly reduces the risk of myocardial infarction under the well-matched condition, which is a dependable choice for patients with carotid stenosis.

## Introduction

One of the pivotal causes of stroke is carotid stenosis (CS) (1). About 30% of ischemic stroke is triggered by extracranial CS, and atherosclerosis occupies 90% of adverse lesions leading to ischemia (2). At present, a long-term global survey indicated that stroke, as a severe disease threatening human life, has become the second leading cause of death and the third of disability (3). Elderly people are particularly more likely to be subject to stroke (4), due to complex comorbidities or vasculopathy (5). As a consequence, early intervention is necessary for CS to prevent stroke and maintain life quality of patients.

Several randomized controlled trials have demonstrated the safety and efficacy of transfemoral carotid artery stenting (TF-CAS) and carotid endarterectomy (CEA) for symptomatic or asymptomatic CS (6–9). However, TF-CAS has commonly been considered as an alternative to CEA, which is seen as the gold standard for treating CS (10–12). Even though TF-CAS has reached equivalent effects on late outcomes as CEA (13) and has been more favorable for patients with higher risks at anatomy or clinical picture (14). However, during the perioperative period, TF-CAS is associated with a greater risk of neurological events, which has been documented in quite a few studies (15–17). The critical reasons resulting in the failure for TF-CAS to optimize outcomes include inconvenient manipulation for the guidewire to pass through the aortic arch, and plaque fracture or thrombus embolizing intracranial arteries when carrying protected device through carotid lesion (18). At this stage, a newer technique (transcarotid artery revascularization, TCAR) has been valued and developed rapidly, which consists of direct manipulation to the lesion and minimized incision with short-path stenting (19). Moreover, as an assisted neuroprotection device, the flow reversal system significantly improves the efficacy of TCAR, which is extracorporeal arteriovenous access and filters bloodstream into the brain (20, 21).

Recently, a meta-analysis has demonstrated the short-term and long-term efficacy and safety of TCAR (22). Moreover, two prospective, single-arm, multicenter studies (ROADSTER and ROADSTER2) have also shown that TCAR was associated with satisfactory outcomes, such as the rates of freedom from stroke, transient ischemic attack, and death in the perioperative period after the procedure (23, 24). In the context of such favorable postoperative prognoses of TCAR, comparative studies were performed compared to conventional therapies (25–27), and this emerging technique also partly presented superiority over the transfemoral procedure. A 2019 meta-analysis found that the transcarotid approach reduced the risk of stroke contrasted with TF-CAS (28). In addition, high-volume, multicenter research using the propensity score matching (PSM) suggested TCAR with dynamic flow reversal had significantly mitigated the rate of stroke/death than TF-CAS (29). Being the first-class choice for CS, it is inevitable for CEA to be a reference standard measuring this novel technique. However, few randomized controlled trials comparing TCAR and CEA were conducted, which means it is still a lack of high-level evidence that TCAR may be comparable to CEA for CS. Although a 2020 meta-analysis comprised of non-randomized studies indicated that the two procedures were equivalent on stroke/death/myocardial infarction (MI), the less power was limited to the relatively small sample size of the TCAR arm (30). In addition, the comparative effects based on different symptom status remain uncertain. Given these margins in current meta-analyses, a complementary study was carried out by us, which aims to explore the efficacy of TCAR compared to CEA in a larger sample size, especially for symptomatic patients.

## Materials and Methods

This systematic review and meta-analysis have reported items according to the PRISMA statement to complete a specific project mainly, such as article retrieval (31), data synthesis, and integrated analysis.

### Search Strategy

**Figure 1:**
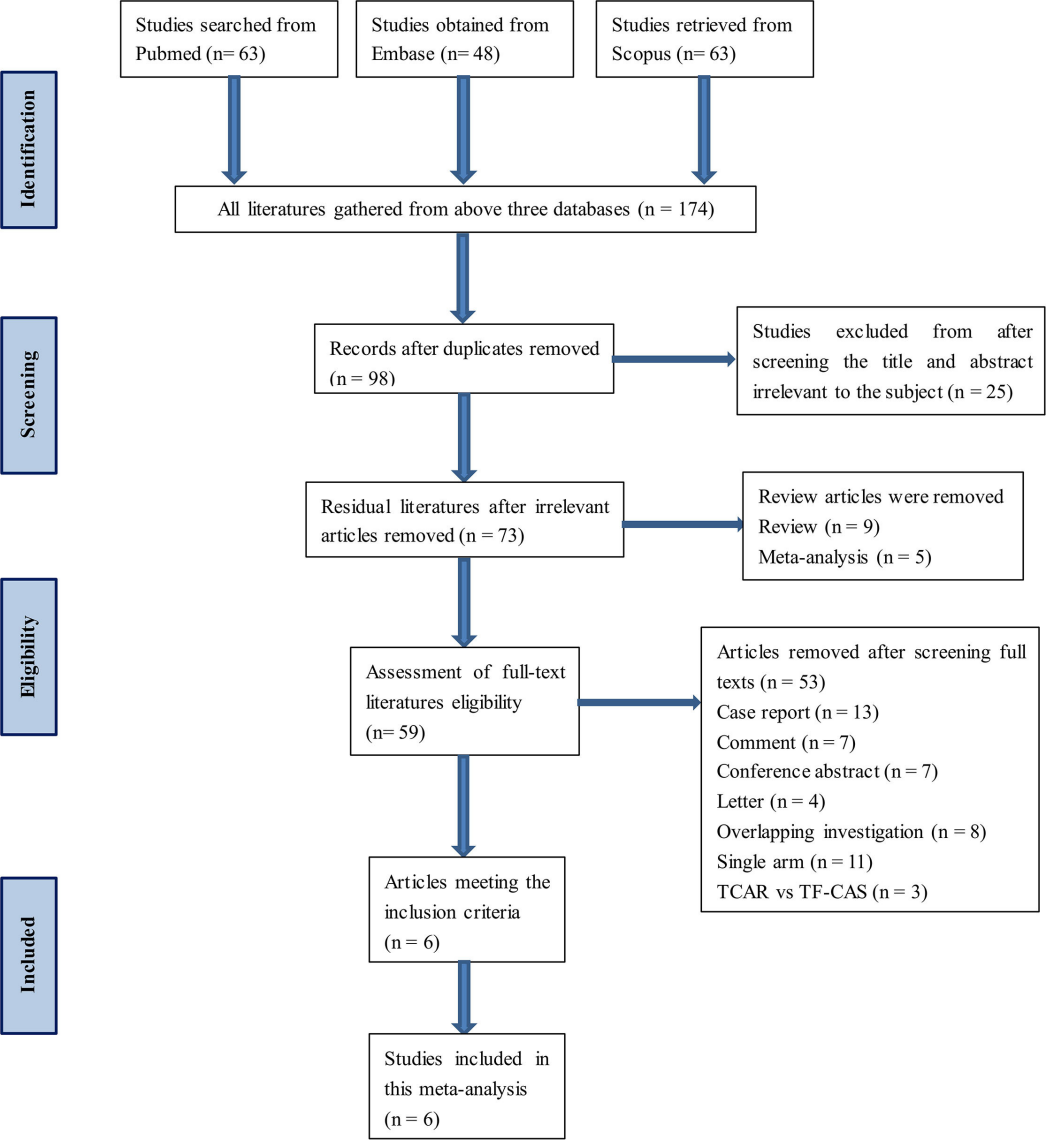
Flow diagram of search results and article screening.

Databases, namely, PubMed, Embase, and Scopus, were searched and no literature was added from other channels. The keywords contained TCAR, transcervical, CEA, and CS. This process was carried out by two independent investigators. All the selected works of literature meet the inclusion criteria. Besides, any disagreement during the inclusion process would be reviewed and resolved by the third researcher. A detailed flow diagram of articles screening is presented in Figure 1. Search strategy within each database can be found in Supplementary Table 1.

### Selection Criteria

The study to be included needs to meet all the following criteria: (1) the type of research was comparative analysis, (2) perioperative medication was presented in the study, (3) TCAR vs. CEA, and (4) relevant endpoints of interest. And works of literature that satisfied any of the following criteria were excluded: (1) non-clinical trial, (2) the use of flow reversal system in TCAR group was not described, (3) symptomatic status was not reported in each cohort, (4) conference abstract or case report, (5) TCAR vs. TF-CAS, and (6) overlapping investigation (data of patients from the same available sites may be repeatedly used in multiple studies).

### Data Extraction and Endpoints

Detailed contents, such as publication time, study type, enrollment period, and inclusion and exclusion criteria, in each eligible literature, were collected (as shown in Table 1). The primary endpoint was postoperative stroke/death/MI. Death, stroke, MI, hemorrhage, cranial nerve injury (CNI), and procedure time were classified as secondary outcomes within 30 days after the procedure. Clinical endpoints concerning overall, symptomatic, or asymptomatic cohort were also extracted respectively.

**Table 1:** Characteristics of included studies.

**Study**	**Type and source**	**Enrollment period**	**Inclusion criteria**	**Exclusion criteria**
Alvarez et al. (32)	Retrospective Comparative 1 center	TCAR: 2005–2007 CEA: 2002–2005	Age ≥ 80 years CS ≥ 70%	NA
Plessers et al. (33)	NA 1 center	NA	SCS ≥ 60% ACS ≥ 80%	Ostial CCA or tandem lesions
Kashyap et al. (34)	Retrospective Comparative 4 centers	2013–2017	SCS ≥ 50% ACS ≥ 70%	NA
Malas et al. (35)	Prospective Comparative TCAR: 296 centers CEA: 369 centers	2016–2019	No concomitant procedure	Tandem, traumatic or dissection lesions >1 stented lesion unknown symptom status planned intracranial procedures
Yee et al. (36)	Retrospective Comparative 1 center	2011–2018	Not registered in ROADSTER2 trial	Tandem intracranial stenosis, arteriovenous malformation, tumor
Cappellini et al. (37)	Retrospective Comparative 1 center	TCAR: 2018–2019CEA: 2012–2019	SCS ≥ 50% ACS ≥ 80% CCA diameter ≥ 6 mm Length of CCA to bifurcation ≥ 5 cm	NA

*CS, carotid stenosis; SCS, symptomatic carotid stenosis; ACS, asymptomatic carotid stenosis; TCAR, transcarotid artery revascularization; CEA, carotid endarterectomy; NA, not available; CCA, common carotid artery*.

### Quality Assessment and Risk of Bias

Newcastle-Ottawa Scale was used for quality assessment of each comparative study (38), which included three aspects: selection, comparability, and outcomes. According to the scoring system, the full score is 9. When the study scored 1–4 is classified as low quality articles methodologically, 5–7 as moderate quality, and 8–9 as high. The risk of bias and certainty of findings were described according to the grading system (39) designed by the grades of recommendation, assessment, development, and evaluation (GRADE) working group.

### Data Synthesis and Heterogeneity

Review manager 5.4 was used for statistical analysis. Odds ratio (OR) and mean difference in 95% CI were selected as the effect size to reflect prognosis undergoing different treatment. *P* < 0.05 indicated that results were statistically significant. Fixed effects model was the preference unless any heterogeneity determined by I^2^ statistic was found, if so, random-effects model was carried out to adjust the ORs. According to the Cochrane handbook, the heterogeneous level was sorted as low, moderate, substantial, and considerable corresponding to *I*^2^ <40%, 30–60%, 50–90%, and 75–100%, respectively (40). Sensitivity analyses were performed to identify the stability of results (41).

### Definitions

1. Symptomatic status was defined as having a transient ischemic attack, amaurosis fugax, or stroke in the previous 180 days.
2. Stroke was defined as ipsilateral or contralateral, cortical or vertebrobasilar, and ischemic or hemorrhagic strokes. If the symptoms last less than 24 h would be considered as a transient ischemic attack.
3. Myocardial infarction was defined as acute clinical symptoms plus troponin significantly increased or electrocardiogram sharp changed.
4. Hemorrhage was defined as hematoma requiring surgery or intervention.

## Results

### Characteristics of the Included Studies

A total of 174 articles were obtained through the retrieval flow, thereafter removing duplicates and screening abstracts or full texts, the remaining six comparative cohort research studies were finally selected in this meta-analysis (32–37). Among all excluded studies, an overlapping investigation might occur in eight trials. TCAR group and CEA group were individually comprised of 6,881 and 7,319 patients with CS. The average age of patients in the TCAR cohort was slightly older than the CEA group, but no statistical significance was shown. The prevalence distributions of hypertension, diabetes, hyperlipidemia, and coronary artery disease were not discrepant in each study except for Cappellini et al. (37) containing more patients with coronary artery disease in the TCAR arm. Most subjects treated by the transcarotid approach were given general anesthesia, but Plessers et al. (33) reported that only 25% was received. Five included articles indicated that procedure time could be shortened by transcarotid way, which was significantly less than endarterectomy. Only Yee et al. documented intra-operative blood loss and the outcome favored TCAR. Dynamic flow reversal system had to be attached to TCAR technique in principle if there is no severe intolerance or contraindication. More preoperative and intra-operative information are shown in Table 2.

**Table 2:** Preoperative and intraoperative information of patients with carotid stenosis.

	**Alvarez et al.** **(****32****)**		**Plessers et al.** **(****33****)**		**Kashyap et al.** **(****34****)**		**Malas et al.** **(****35****)**		**Yee et al.** **(****36****)**		**Cappellini et al.** **(****37****)**	
	**TCAR**	**CEA**	**TCAR**	**CEA**	**TCAR**	**CEA**	**TCAR**	**CEA**	**TCAR**	**CEA**	**TCAR**	**CEA**
Patient (*n*)	36	45	16	10	292	292	6,384	6,384	87	87	66	501
Age	83.5 ± 3.35	81.7 ± 1.55	71.3 ± 9.5	65.7 ± 4.3	71.1 ± 8.7	70.9 ± 8.5	74	74	71.1 ± 9.1	71.6 ± 10.1	74.3 ± 9.5	71.6 ± 9.6
Male	31 (86.1)	36 (80)	13 (81.3)	6 (60)	(65.8)	(63.0)	4,070 (63.8)	4,078 (63.9)	(85.1)	(88.5)	(65.2)	(62.5)
Hypertension	30 (83.3)	32 (71.1)	11 (68.8)	6 (60)	(91.1)	(91.1)	5,791 (90.7)	5,853 (91.7)	(88.5)	(92.0)	(92.4)	(89.8)
Diabetes	11 (30.6)	15 (33.3)	5 (31.3)	3 (30)	(45.9)	(40.4)	2,451 (38.4)	2,540 (39.8)	(50.6)	(47.1)	(39.4)	(35.5)
Hyperlipidemia	16 (44.4)	15 (33.3)	15 (93.8)	10 (100)	(82.9)	(74.4)	5,708 (89.4)	5,755 (90.1)	(83.9)	(93.1)	—	—
CAD	16 (44.4)	10 (22.2)	—	—	(53.4)	(45.9)	3,066 (48.0)	3,042 (47.6)	(51.7)	(51.7)	(75.8)	(17.4)
Smoking	3 (8.3)	2 (4.4)	—	—	(59.6)	(67.8)	1,391 (21.8)	1,433 (22.4)	(33.3)	(26.4)	(18.2)	(21)
Symptomatic	11 (30.6)	20 (44.4)	8 (50)	6 (60)	(35.3)	(36.3)	1,658 (26.0)	1,675 (26.2)	(44.8)	(50.6)	(54.5)	(52.2)
**Intraoperative**												
General anesthesia	36 (100)	45 (100)	4 (25)	10 (100)	(93.8)	(96.9)	5,356 (83.9)	5,418 (84.9)	(98.9)	(98.9)	(97.0)	(100)
Mean time (*min*)	—	—	76.5	101.9	74.9	109.4	72.5	121.4	73.2	144.1	57.4	105.2

*Data were given as mean ± SD, n, or n (%). CAD, coronary artery disease; TCAR, transcarotid artery revascularization; CEA, carotid endarterectomy*.

According to our independent adjudication, four of six works of literature were moderate quality and the other two were high quality by the NOS scale (Table 3). In terms of comparability of cohorts, Kashyap et al. (34), Malas et al. (35), and Yee et al. (36) have adjusted critical confounders containing age, gender, symptom status, hyperlipidemia, diabetes, and hypertension. Either age and gender or common comorbidities were balanced against between two arms in the remaining articles (32, 33, 37). Moreover, being a feasible and recommended method, PSM improves intergroup comparability and was also applied in the two works of literature by Kashyap et al. (34) and Malas et al. (35), respectively.

**Table 3:** Quality assessment of comparative studies based on Newcastle-Ottawa Scale.

**Criterion**	**Alvarez et al. (32)**	**Plessers et al. (33)**	**Kashyap et al. (34)**	**Malas et al. (35)**	**Yee et al. (36)**	**Cappellini et al. (37)**
**Selection of cohorts**						
Representativeness of exposed cohort (TCAR)	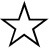	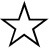	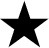	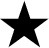	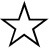	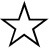
Selection of non-exposed cohort (CEA)	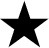	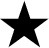	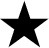	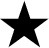	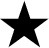	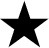
Ascertainment of exposure	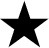	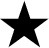	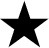	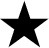	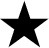	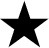
Demonstration that outcome of interest was not present at start of study	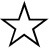	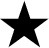	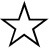	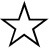	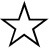	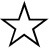
**Comparability**						
Comparability of cohorts by design or analysis	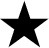	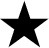	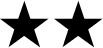	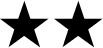	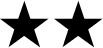	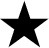
**Assessment of outcomes**						
Assessment of outcomes	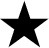	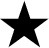	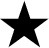	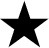	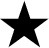	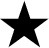
Was follow up long enough (at least 30 days)	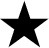	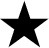	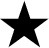	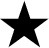	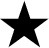	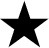
Adequacy of follow up (≥95%)	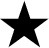	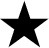	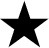	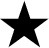	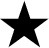	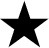
**Total score**	6	7	8	8	7	6

*The full score is 9 based on the Newcastle-Ottawa scoring system. Low quality: 1–4, moderate quality: 5–7, high quality: 8–9. TCAR, transcarotid artery revascularization; CEA, carotid endarterectomy*.

### Outcomes of Data Synthesis

#### Stroke/Death/MI

Three research studies have reported concerning data based on mentioned definitions (6,712 TCARs among 13,433 patients). The composite incidence in TCAR and CEA group was 2.0% and 2.4% individually without statistical significance (OR 0.85, 95% CI 0.67–1.07, *P* = 0.17). No heterogeneity was detected (I^2^ = 0%; Supplementary Figure 1A).

#### Death

Data were extracted from six studies (6,881 TCARs among 14,200 patients). Slightly higher mortality in the TCAR cohort was 0.4% compared to 0.3% of that for CEA within 30 days after the procedure. However, there is no significant difference between the two approaches and no heterogeneity in pooled analysis (OR 1.14, 95% CI 0.67–1.94, *P* = 0.63, I^2^ = 0%; Supplementary Figure 1B).

#### Stroke

Although all articles have reported postoperative stroke, Yee et al. was excluded from data synthesis, because the definition may include transient ischemic attack. A total of 6,794 and 7,232 patients underwent TCAR and CEA respectively, 1.4% compared to 1.3% of stroke rates, which is a non-heterogeneous result with no statistical discrepancy (OR 1.03, 95% CI 0.77–1.37, *P* = 0.84, I^2^ = 0%; Supplementary Figure 1C).

#### Myocardial Infarction

Postoperative incidence of MI was available from five works of literature (6,865 TCARs among 14,174 patients). The significant difference indicated the MI rates in the TCAR arm was 0.5% contrasted with 0.9% in controls (OR 0.55, 95% CI 0.36–0.83, *P* = 0.004, I^2^ = 0%; Supplementary Figure 1D).

#### Hemorrhage

Four studies have reported this endpoint (6,573 TCARs among 13,590 patients). TCAR was not differed from CEA statistically in terms of reducing the risk of hemorrhage and no heterogeneity was found (OR 0.77, 95% CI 0.58–1.03, *P* = 0.08, I^2^ = 0%), however, in fact, the transcarotid approach had a lower 0.4% of risk degree (CEA: 1.7%, TCAR: 1.3%; Supplementary Figure 1E).

#### Cranial Nerve Injury

Data regarding CNI were available from all research studies (6,881 TCARs among 14,200 patients). This non-heterogeneous result favored that TCAR was strongly associated with a much lower risk of CNI after the procedure than CEA (0.3 vs. 1.4%, OR 0.22, 95% CI 0.14–0.36, I^2^ = 0%), which was as same as all the included articles (Supplementary Figure 1F).

#### Procedure Time

The finding based on five studies suggested that TCAR had significantly shortened operative time compared to CEA (6,867 TCARs among 14,475 patients), though the heterogeneity was considerable (Supplementary Figure 1G).

### Subgroup Analysis

Detailed information of pooled analysis based on symptomatic or asymptomatic status is recorded in Table 4, and corresponding forest plots are shown in Supplementary Figures 2, 3 individually.

**Table 4:** Subgroup analysis of symptomatic or asymptomatic status for carotid stenosis treated by TCAR or CEA.

	**Symptomatic**					**Asymptomatic**				
**Outcomes**	**No. of studies**	**TCAR**	**CEA**	***P***	***I*** ^**2**^	**No. of studies**	**TCAR**	**CEA**	***P***	***I*** ^**2**^
Stroke/death/MI	2	2.7	3.5	0.18	0	2	1.8	2.0	0.47	0
Stroke	2	2.1	2.2	0.85	0	2	1.2	1.1	0.7	0
Death	2	0.6	0.6	0.9	0	2	0.3	0.3	0.84	0
MI	2	0.5	1.0	0.07	0	2	0.5	0.9	0.54	27
CNI	2	0.3	3.1	SS	11	2	0.3	2.6	SS	0

*MI, myocardial infarction; CNI, cranial nerve injury; TCAR, transcarotid artery revascularization; CEA, carotid endarterectomy; SS, statistically significant. Data in the column of TCAR and CEA were given as percent (%)*.

#### Symptomatic Status

Pre-designed subgroup analyses were performed to study the comparative effects between both approaches in different symptom status. Kashyap et al. and Malas et al. have divided subjects into two arms according to the mentioned definition. Among the symptomatic cohort, no statistical significance was found between the two groups in terms of the primary endpoint, stroke, death, or MI. However, TCAR was correlated to the decline of CNI and statistically superior to CEA under the random-effects model (OR 0.12, 95% CI 0.04–0.32, *P* < 0.0001, I^2^ = 11%).

#### Asymptomatic Status

The investigation concerning asymptomatic patients has also presented that no significant discrepancies were detected on stroke, death, or MI.

Although the incidence of postoperative MI in TCAR troop was similar to that in CEA, a little heterogeneity was shown in the plot (OR 0.71, 95% CI 0.24–2.10, *P* = 0.54, I^2^ = 27%). Besides, the non-heterogeneous result indicated that the higher CNI rate still occurred in the CEA cohort, with statistical significance.

### Sensitivity Analysis

All studies at each endpoint would be single removed to verify the stability of the results. Severe instabilities containing reversed statistical significance or obvious changes in effect estimates were shown below.

#### Myocardial Infarction

After eliminating the research of Malas et al., in spite of heterogeneity was also not detected, the statistical significance favored TCAR disappeared, which means TCAR has no longer superior to CEA in terms of MI for the overall cohort (OR 0.70, 95% CI 0.17–2.90, *P* = 0.63, I^2^ = 0%).

#### Hemorrhage

Even though removing the study by Malas et al. will lead to a decline of the OR from 0.77 to 0.38, the non-significant difference still exists between the two therapies for entire subjects (95% CI 0.08–1.83, *P* = 0.23, I^2^ = 0%).

## Discussion

About one-third of healthy looking elder people suffered from atherosclerotic CS (42). With the development of plaque burden, the lumen patency to the brain is directly affected, in addition, the fractured fragments or thrombus may severely obstruct the neurological arteries and cause ischemic stroke. As the two effective therapies used for CS frequently, TF-CAS and CEA are unavoidable topics, however, the latter has most likely to be recommended due to the lower risk of stroke (43). Nowadays, a novel technique composed of carotid stenting and specially designed device was seen as a third strategy for treating CS, which received positive comment and efficacy, and distinctly reduced adverse events within the perioperative period (44–46). Therefore, it is necessary to study the prognosis of patients who underwent TCAR compared to CEA in a larger sample size and to explore whether there are differences related to symptom status.

From our findings, of all the patients in the TCAR cohort, in terms of stroke/death/MI, death, stroke, or hemorrhage, which is 2.0, 0.4, 1.4, and 1.3% respectively, is similar to the CEA cohort. In addition, the rates of adverse events are equivalent to the results reported in the current literature (23, 29, 47). Only a few included studies have presented a slightly higher stroke rate for TCAR compared to CEA, though this may due to patient selection or the relatively small sample size (34). The reduction of neurological risks from TF-CAS to TCAR, to a great extent, is associated with the use of flow reversal, which is a protected device driving thrombus to femoral vein based on arterio-venous pressure, meanwhile, directly carotid puncture avoiding manipulation in the aortic arch is another beneficial factor (48, 49).

It is worth noting that patients who underwent CEA were mostly like to be subject to MI than the TCAR group, which is identical to several published studies (35, 50). Considering the adjustment by PSM, even the prior health condition of subjects was comparable between two groups, the edge of TCAR over CEA is not difficult to explain. This situation maybe resulted from not only shorter procedure time and small transcervical incision, which reduces intra-operative blood loss and declines the burden on cardiopulmonary function, but also less use of general anesthesia and more active postoperative medications in the TCAR arm compared to CEA. On the other hand, in view of patients treated by TCAR could be discharged hospital generally earlier than CEA, potentially, which is an inconvenient plight for whole centers to capture the changes of ST-segment through electrocardiograph timely when asymptomatic MI happened, and imaging examination may be ignored due to the subjects were freedom from symptoms in the follow-up stage. Surprisingly, through the sensitivity analysis of MI for the overall cohorts, after removing the well-matched study with a large sample size by Malas et al., the statistical difference in favor of TCAR is lost. This study occupies 92.3% of total weight and suggests the MI rate among TCAR arm notably lower than CEA arm, however, the pooled data from residual studies showed equivalent efficacy, even the physical function was worse in the TCAR group. In other words, there is no statistical importance but a clinical value. Within a certain risk range, for patients under similar challenges from both procedures, TCAR might be more beneficial and safer. Despite no significant difference in MI was found in either symptomatic or asymptomatic cohort, which is possibly limited to the nature of PSM (51) where a part of patients who underwent CEA was excluded, hence the statistical significance was not revealed.

Symptomatic patients tend to be more sensitive to cerebral ischemia and also benefit apparently on postprocedure recovery of physical function from carotid surgery (52, 53). As shown in this analysis, TCAR has reached comparable performance to CEA on preventing stroke for symptomatic carotid stenosis (SCS), without significance, but the OR slightly supported the former. For SCS, being the reliable treatment demonstrated by the NASCET trial (54), however, accompanied by increasing age, CEA had been proven associated with higher perioperative risks, especially stroke or death (55). Besides, a current study comparing CEA with TCAR suggested that TCAR has reduced adverse events for symptomatic patients aged over 80 (56), this mostly owing to the active anesthesia management that maintains the steady state of hemodynamics, but also the appropriate lesions meeting the specific anatomy criteria, such as common carotid artery diameter ≥6 mm, clavicle to bifurcation ≥5 cm, and no atherosclerotic plaque at the access site, which optimize patient selection and bring the CEA-risk better prognoses through diminishing procedure threshold.

Although the management of asymptomatic carotid stenosis (ACS) has always been controversial (57), CEA has still been recommended by guidelines due to the considerable prognosis resulted from multi-center randomized trials (58, 59). According to our findings, TCAR is identical to CEA for ACS in terms of reducing stroke, death, or MI, except for CNI, which is irrespective of symptom status but related to the nature of CEA. Malas et al. have indicated the two therapies were statistically equivalent on postoperative death after matching the baselines (35), and similar results were also reported by other two literature (44, 50). From the incidence perspective in this meta-analysis, the TCAR cohort had shown slightly higher mortality, but no statistical difference was detected. In summary, it is reasonable to speculate that the TCAR with flow reversal has non-inferiority to CEA for ACS.

In this pooled study, the certainty of evidence was assessed as moderate and low due to the risk of bias (Table 5). The confounding bias of included studies was generally limited to the inherent traits of non-randomized trial downgrading the level of evidence, moreover, various stenotic degrees, lesion length, and anesthesia details will decline the applicability of findings. However, given the higher prevalence of medical comorbidities in the TCAR arm will negatively affect the effects, in order to reflect the authentic situation, the certainty needed to be upgraded. Meanwhile, according to the Grade scoring tool, consistent evidence concerning CNI can also be upgraded due to the magnitude of the large effect. Additionally, here the funding information or commercial support potentially led to reporting bias was investigated in this analysis. The research of Plessers et al. was supported by scientific funding from Belgium (33). Although these three studies have reported no funding obtained (34–36), at least one author in each article has ever received sponsorship or served as a consultant in the Silk Road Medical, which is an institution responsible for researching and developing TCAR with flow reversal system. The residual two literature have none of the above situations (32, 37).

**Table 5:** Summary of findings and bias analysis for included studies on each prognostic indicator.

**Outcomes**	**Participants (studies)**	**Risk of bias**	**Inconsistency**	**Indirectness**	**Imprecision**	**Anticipated absolute effects (95% CI)**		**Relative effect (95% CI)**	**Certainty**
						**Risk with CEA**	**Risk with TCAR**		
Stroke/death/MI	13,433 (3 cohort studies)	serious	not serious	not serious	not serious	24 per 1,000	20 per 1,000	OR 0.85 (0.67 to 1.07)	⊕⊕◯◯^a^ LOW
Death	14,200 (6 cohort studies)	serious	not serious	not serious	not serious	4 per 1,000	4 per 1,000	OR 1.14 (0.67 to 1.94)	⊕⊕◯◯^a^ LOW
Stroke	14,026 (5 cohort studies)	serious	not serious	not serious	not serious	13 per 1,000	14 per 1,000	OR 1.03 (0.77 to 1.37)	⊕⊕◯◯^a^ LOW
MI	14,174 (5 cohort studies)	serious	not serious	not serious	not serious	9 per 1,000	5 per 1,000	OR 0.55 (0.36 to 0.83)	⊕⊕◯◯^a^ LOW
Hemorrhage	13,590 (4 cohort studies)	serious	not serious	not serious	not serious	17 per 1,000	13 per 1,000	OR 0.77 (0.58 to 1.03)	⊕⊕◯◯^a^ LOW
CNI	14,200 (6 cohort studies)	serious	not serious	not serious	not serious	14 per 1,000	3 per 1,000	OR 0.22 (0.14 to 0.36)	⊕⊕⊕◯^a^^.^^b^ MODERATE

*CI, Confidence interval; OR, Odds ratio; MI, Myocardial infarction; CNI, Cranial nerve injury; TCAR, Transcarotid artery revascularization; CEA, Carotid endarterectomy*.

a *: The plausible confounders would reduce the effect of TCAR*.

b *: The magnitude of a large effect will upgrade the quality of evidence*.

As a result of a randomized trial comparing TCAR with CEA is still lacking (60), no powerful evidence could be found to support our findings. Even so, this meta-analysis has demonstrated that the perioperative efficacy of TCAR is similar to CEA under a larger sample size and also compared the two therapies in different symptom status, which are steps forward building on the foundation of the previous study (30).

### Limitations

Firstly, no randomized controlled trial could be obtained during searching databases, only several cohort studies met pre-specified inclusion criteria, which have increased the risk of bias and weakened evidence. Besides, the definition of stroke in the research of Yee et al. (36) includes transient ischemic attack, hence, after eliminating it, the decline of sample size would affect OR. No economic data were available so that could not be analyzed in this study to guide clinical choice. Indeed, PSM may lead to selection bias, and low-risk patients who underwent CEA were excluded, however, it vastly balanced the confounders between two cohorts and improved comparability. Although a pooled analysis was not performed due to absent data, prior investigation presented the learning curve of TCAR is short, even in the novice stage, the procedures can be completed with lower stroke or death (61). Lastly, considering the lack of patient-level details of anesthesia, which type of CS undergoing TCAR can obtain a better prognosis from local or general anesthesia remains unknown in this meta-analysis.

## Conclusions

From this meta-analysis, TCAR has achieved comparable efficacy to CEA on preventing stroke/death/MI, stroke, death, and reached better in terms of CNI and operation time, irrespective of symptom status. Under the well-matched condition, TCAR can more likely reduce MI rate than CEA. More high-volume, prospective and long-term comparative studies are needed to testify our findings.

## Data Availability Statement

The original contributions presented in the study are included in the article/Supplementary Material, further inquiries can be directed to the corresponding author.

## Author Contributions

JG: data collection and synthesis analysis. JG and ZC: quality assessment of included literatures. ZC, LK, HZ, and YY: manuscript reversion. JG, ZC, LK, HZ, and YY: final approval of the article. All authors contributed to the article and approved the submitted version.

## Conflict of Interest

The authors declare that the research was conducted in the absence of any commercial or financial relationships that could be construed as a potential conflict of interest.

## Publisher's Note

All claims expressed in this article are solely those of the authors and do not necessarily represent those of their affiliated organizations, or those of the publisher, the editors and the reviewers. Any product that may be evaluated in this article, or claim that may be made by its manufacturer, is not guaranteed or endorsed by the publisher.
