# Supplementary material for: The Efficacy of Transcarotid Artery Revascularization With Flow Reversal System Compared to Carotid Endarterectomy: A Systematic Review and Meta-Analysis
Source: Front Cardiovasc Med. 2021 Nov 19;8:695295. doi: 10.3389/fcvm.2021.695295 (PMC8640218; doi:10.3389/fcvm.2021.695295)
Supplement: Supplementary file 3 [file Data_Sheet_3.docx]

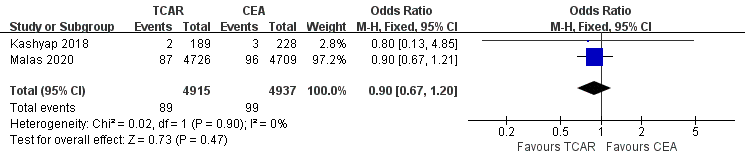


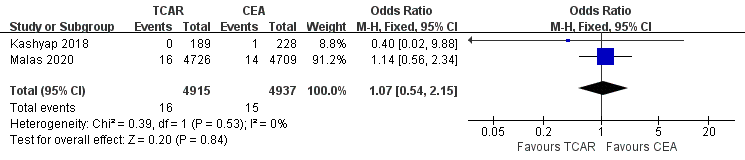


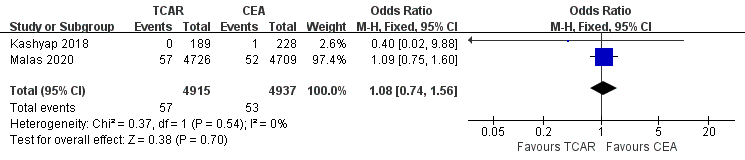


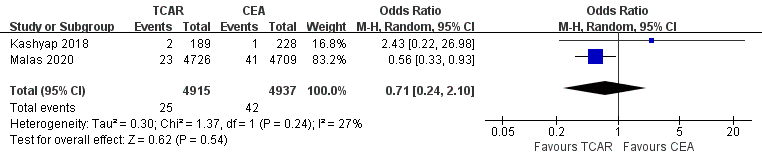


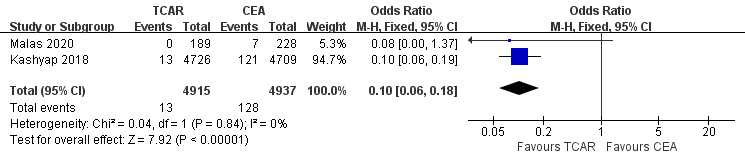


**Supplemental figure 3.** Comparative efficacy between the two approaches on carotid stenosis within asymptomatic cohort. Forest plots of perioperative outcomes containing **(A)** stroke/death/MI, **(B)** death, **(C)** stroke, **(D)** MI, **(E)** CNI for patients with carotid stenosis treated by transcarotid artery revascularization (TCAR) versus carotid endarterectomy (CEA) among asymptomatic cohort.

MI: myocardial infarction, CNI: cranial nerve injury, CI: confidence interval

M-H: Mantel-Haenszel.
