# Supplementary material for: The Efficacy of Transcarotid Artery Revascularization With Flow Reversal System Compared to Carotid Endarterectomy: A Systematic Review and Meta-Analysis
Source: Front Cardiovasc Med. 2021 Nov 19;8:695295. doi: 10.3389/fcvm.2021.695295 (PMC8640218; doi:10.3389/fcvm.2021.695295)
Supplement: Supplementary file 4 [file Table_1.docx]

| **Search strategy - Pubmed** | |
| --- | --- |
| **Items** | **Terms** |
| TCAR | (((TCAR[Title/Abstract]) OR (transcarotid artery revascularization[Title/Abstract])) OR (transcarotid[Title/Abstract])) OR (transcervical[Title/Abstract])) OR (transcarotid revascularization[Title/Abstract]) |
| *and* | |
| CEA | ("Endarterectomy, Carotid"[Mesh]) OR (((Carotid Endarterectomy[Title/Abstract]) OR (Carotid Endarterectomies[Title/Abstract])) OR (Endarterectomies, Carotid[Title/Abstract])) |
| *and* | |
| CS | ("Carotid Stenosis"[Mesh]) OR (((((((((Carotid Stenoses[Title/Abstract]) OR (Artery Narrowing, Carotid[Title/Abstract])) OR (Carotid Artery Stenosis[Title/Abstract])) OR (Internal Carotid Artery Stenosis[Title/Abstract])) OR (Common Carotid Artery Stenosis[Title/Abstract])) OR (External Carotid Artery Stenosis[Title/Abstract])) OR (Artery Plaque, Carotid[Title/Abstract])) OR (Carotid Ulcer[Title/Abstract])) OR (Carotid Artery Ulcerating Plaque[Title/Abstract])) |

| **Search strategy - Embase** | |
| --- | --- |
| **Items** | **Terms** |
| TCAR | 'transcarotid artery revascularization'/exp OR 'transcarotid access'/exp OR 'transcervical approach'/exp OR tcar:ab,ti |
| *and* | |
| CEA | 'carotid endarterectomy'/exp OR 'endarterectomies, carotid':ab,ti OR 'carotid endarterectomies':ab,ti |
| *and* | |
| CS | 'carotid stenosis':ab,ti OR 'artery narrowing, carotid':ab,ti OR 'carotid artery stenosis':ab,ti OR 'carotid artery ulcerating plaque':ab,ti OR 'carotid artery obstruction'/exp |

| **Search strategy - Scopus** | |
| --- | --- |
| **Items** | **Terms** |
| TCAR | ( TITLE-ABS-KEY ( "transcarotid artery revascularization" ) OR TITLE-ABS-KEY ( "transcarotid approach" ) OR TITLE-ABS-KEY ( "transcervical" ) OR TITLE-ABS-KEY ( "TCAR" ) ) |
| *and* | |
| CEA | ( TITLE-ABS-KEY ( "carotid endarterectomy" ) OR TITLE-ABS-KEY ( "carotid endarterectomies" ) OR TITLE-ABS-KEY ( "CEA" ) ) |
| *and* | |
| Continued | |
| CS | ( TITLE-ABS-KEY ( "carotid stenosis" ) OR TITLE-ABS-KEY ( "carotid stenoses" ) OR TITLE-ABS-KEY ( "carotid artery obstruction" ) OR TITLE-ABS-KEY ( "artery narrowing, carotid" ) OR TITLE-ABS-KEY ( "carotid artery ulcerating plaque" ) ) |

**Supplemental Table 1.** Detailed search strategies.
